# Genomic Selection for Grain Yield in the CIMMYT Wheat Breeding Program—Status and Perspectives

**DOI:** 10.3389/fpls.2020.564183

**Published:** 2020-09-15

**Authors:** Philomin Juliana, Ravi Prakash Singh, Hans-Joachim Braun, Julio Huerta-Espino, Leonardo Crespo-Herrera, Velu Govindan, Suchismita Mondal, Jesse Poland, Sandesh Shrestha

**Affiliations:** ^1^ International Maize and Wheat Improvement Center (CIMMYT), Texcoco, Mexico; ^2^ Campo Experimental Valle de Mexico, Instituto Nacional de Investigaciones Forestales, Agricolas y Pecuarias (INIFAP), Chapingo, Mexico; ^3^ Wheat Genetics Resource Center, Department of Plant Pathology, Kansas State University, Manhattan, KS, United States

**Keywords:** wheat, genomic selection, grain yield, quantitative trait, climate-resilience

## Abstract

Genomic breeding technologies offer new opportunities for grain yield (GY) improvement in common wheat. In this study, we have evaluated the potential of genomic selection (GS) in breeding for GY in wheat by modeling a large dataset of 48,562 GY observations from the International Maize and Wheat Improvement Center (CIMMYT), including 36 yield trials evaluated between 2012 and 2019 in Obregón, Sonora, Mexico. Our key objective was to determine the value that GS can add to the current three-stage yield testing strategy at CIMMYT, and we draw inferences from predictive modeling of GY using 420 different populations, environments, cycles, and model combinations. First, we evaluated the potential of genomic predictions for minimizing the number of replications and lines tested within a site and year and obtained mean prediction accuracies (PAs) of 0.56, 0.5, and 0.42 in Stages 1, 2, and 3 of yield testing, respectively. However, these PAs were similar to the mean pedigree-based PAs indicating that genomic relationships added no value to pedigree relationships in the yield testing stages, characterized by small family-sizes. Second, we evaluated genomic predictions for minimizing GY testing across stages/years in Obregón and observed mean PAs of 0.41, 0.31, and 0.37, respectively when GY in the full irrigation bed planting (FI BP), drought stress (DS), and late-sown heat stress environments were predicted across years using genotype × environment (G × E) interaction models. Third, we evaluated genomic predictions for minimizing the number of yield testing environments and observed that in Stage 2, the FI BP, full irrigation flat planting and early-sown heat stress environments (mean PA of 0.37 ± 0.12) and the reduced irrigation and DS environments (mean PA of 0.45 ± 0.07) had moderate predictabilities among them. However, in both predictions across years and environments, the PAs were inconsistent across years and the G × E models had no advantage over the baseline model with environment and line effects. Overall, our results provide excellent insights into the predictability of a quantitative trait like GY and will have important implications on the future design of GS for GY in wheat breeding programs globally.

## Introduction

Global wheat yields are becoming increasingly vulnerable to the threats posed by changing climates, unpredictable temperatures, reduced precipitation, and adverse agro-climatic events (Trethowan et al., 2002; Trnka et al., 2014; Tack et al., 2015; Zampieri et al., 2017; Hatfield and Dold, 2018). Recognizing the exacerbating effects of climate change on food security (Wheeler and von Braun, 2013) and the tremendous variabilities in yield affecting abiotic and biotic stresses in farmer’s fields over years, the Global Wheat Program at the International Maize and Wheat Improvement Center (CIMMYT) prioritizes breeding for high-yielding climate-resilient wheat lines that have stable grain yield (GY) across a range of environmental conditions (Braun et al., 1992). All the advanced wheat breeding lines at CIMMYT are subjected to extensive three-stage or three-year multi-environment GY testing in a set of managed selection-environments (SEs, optimum, heat and drought stressed environments) at CIMMYT’s primary yield testing site, the Norman E. Borlaug Experimental Research Station, Ciudad Obregón, Sonora, Mexico (27°29′N, 109°56′W). These multi-environment trials facilitate stability assessment of the lines and also help minimize the adverse effects of genotype × environment (G × E) interactions on the progress made from selection (Cooper et al., 1995). However, given the expensive and time-consuming nature of the multi-environment trials, there is a clear need to integrate genomic technologies that can accelerate breeding for GY in wheat.

Genomic selection (GS), a genomics-based selection strategy in which the genomic-estimated breeding values obtained from genome-wide molecular markers are used for the selection of individuals (Meuwissen et al., 2001) has gained burgeoning interest in recent years and is advocated as an approach that can dramatically accelerate genetic gains and change the role of phenotyping in breeding (Heffner et al., 2009; Voss-Fels et al., 2019). While GS for GY in wheat has been evaluated in several studies (Zhao et al., 2013; Charmet et al., 2014; Juliana et al., 2018b; Juliana et al., 2018a; Juliana et al., 2019; Lozada et al., 2019) and models incorporating G × E effects have been reported to increase GY prediction accuracies (PAs) (Crossa et al., 2006; Burgueño et al., 2012; Heslot et al., 2013; Jarquín et al., 2014; Pérez-Rodríguez et al., 2017), a comprehensive evaluation of GY predictions in different stages of yield testing, comparison of genomic PAs with baseline PAs to understand the value of using GS, and a quantitative genetic assessment of GY that can provide insights into genomic PAs are lacking. Hence, we designed this study to determine the value that GS can add to the current three-stage yield testing strategy at CIMMYT, evaluate several GS implementation scenarios for GY that are of importance to wheat breeding programs globally and also explore the prospects of using GS to minimize the number of lines, years, and environments tested by borrowing information from relatives, correlated years and sites.

A large number of GY evaluation datasets at CIMMYT were leveraged for predictive modeling of 48,562 GY observations from 36 yield trials grown between 2012 and 2019 in Obregón, and the key objectives of this study were to (i) evaluate the potential of genomic predictions for minimizing the number of replications and lines tested within a site and year for GY and compare genomic PAs to baseline PAs from the pedigree predictions to understand the relative advantage of genomic relationships over pedigree relationships, (ii) evaluate genomic prediction for GY in full-sib families, understand the relationship between the differences in GY among the full-sibs and the genomic relationships among full-sibs and test the hypothesis if closely related full-sibs had small differences in GY and *vice versa*, (iii) evaluate genomic predictions for minimizing GY testing across stages/years in Obregón using a model with the year, genomic and genotype × year interaction effects (G × E model, where ‘E’ refers to the year) and compare it to a baseline model with only the environment/year and line effects (EL model, where ‘E’ refers to the year), (iv) evaluate genomic predictions for minimizing GY testing in the simulated environments of Obregón (the optimally irrigated, heat and drought stressed environments) using the G × E model and compare the PAs to the PAs from an EL model (‘E’ in both the models refers to the simulated environments), and (v) understand GY predictabilities in relation to quantitative genetic parameters like GY variance components, heritabilities, and phenotypic and genetic correlations across environments.

## Materials and Methods

### Populations, Phenotyping Environments and Best Linear Unbiased Estimates for Grain Yield

We used the following three stages of wheat yield trials from CIMMYT that were evaluated for GY (harvested grain weight calculated on a plot-basis) at Ciudad Obregón as follows:

1) Stage 1 of yield testing or preliminary yield trials: These trials comprised >6,400 in each cycle, that were developed using the selected bulk breeding scheme and were selected visually in earlier generations for agronomic type, phenology, leaf rust, stem rust, stripe rust, fertility, tillering capacity, grain size, and grain health. These lines were evaluated for GY during four crop cycles from 2012–2013 to 2015–2016. Yield potential testing was done in the full irrigation bed planting environment (S1 FI BP), where the lines were grown in two replications on raised beds in an alpha-lattice design during the optimum planting time (third week of November–first week of December) and irrigated optimally with a total of 500 mm water in five irrigations.
2) Stage 2 of yield testing or elite yield trials: These trials comprised lines that were selected from Stage 1 trials for high GY, acceptable agronomic type, heading and maturity, good resistance to rusts, and acceptable to good end-use quality. About 1,092 lines in each cycle were sown in an alpha-lattice design in trials each comprising 28 lines and two high-yielding check varieties in six blocks, during four crop cycles from 2013–2014 to 2016–2017. Yield testing was done in three replications under six managed environments in Obregón that included:
 (i) Full irrigation Bed planting (S2 FI BP)—The lines were planted on raised beds during the optimum time in an optimally irrigated environment that received a total of about 500 mm of water in five irrigations.
 (i) Full irrigation Flat planting (S2 FI FP)—The lines were planted in flat seed beds during the optimum time in an optimally irrigated environment that received a total of about 500 mm of water in five irrigations.
 (i) Reduced irrigation Bed planting (S2 RI)—The lines were planted in raised beds during the optimum time in a reduced irrigation environment that received a total of about 250 mm of water in two irrigations.
 (i) Drought stress Flat planting (S2 DS)—The lines were planted in flat seed beds during the optimum time and grown with a total of about 180 mm of water provided through drip irrigation.
 (i) Early sown heat stress Bed planting (S2 ESHS)—The lines were planted in raised beds during mid-October (about 30 days before the optimum planting time) in an optimally irrigated environment that received a total of about 500 mm of water in five irrigations and evaluated for GY under high-temperature stress during the juvenile growth stage.
 (i) Late sown heat stress Bed planting (S2 LSHS)—The lines were planted in raised beds during the last week of February (about 90 days after the optimum planting time) in an optimally irrigated environment that received about 500 mm of water in five irrigations and evaluated for GY under high-temperature stress during the heading and grain-filling stages.
3) Stage 3 of yield testing or advanced elite yield trials: These trials comprised lines that were selected from the Stage 2 trials for high GY in different environments, agronomic traits, good resistance to rusts, and other foliar diseases and end-use quality. The trial design was similar to that in stage 2 of yield testing, and about 280 lines in each cycle were evaluated in three replications during four crop cycles from 2014–2015 to 2017–2018 in Obregón, under three managed environments that have been described previously: full irrigation bed planting (S3 FI BP), drought stress flat planting (S3 DS), and the late sown heat stress bed planting (S3 LSHS) environments.

The best linear unbiased estimates (BLUEs) for GY in each of the populations, sites, and years were calculated using the ASREML statistical package (Gilmour, 1997), using the following mixed model:

(1) $$y_{ijkl}=\mu +g_{i}+t_{j}+r_{k\left(j\right)}+b_{l\left(jk\right)}+\varepsilon_{ijkl}$$

where *y_ijkl_* is the observed GY, *μ* is the overall mean, *g_i_* is the fixed effect of the entry, *t_j_* is the random effect of the trial that was independent and identically distributed (IID) $\left(t_{j}\sim N\;\left(0,\;\sigma_{t}^{2}\right)\right)$, *r_k_*_(_ *_j_*_)_ is the random effect of the replicate within the trial with IID $\left(r_{k\left(j\right)}\sim N\;\left(0,\;\sigma_{r}^{2}\right)\right)$, *b_l_*_(_ *_jk_*_)_ is the random effect of the incomplete block within the trial and the replicate with IID $\left(b_{m\left(jk\right)}\sim N\;\left(0,\;\sigma_{b}^{2}\right)\right)$and *ε_ijkl_* is the residual with IID $\left(\varepsilon_{ijkl}\sim \;N\;\left(0,\;\sigma_{\varepsilon }^{2}\right)\right)$. Huber’s robust fit outliers method (Huber and Ronchetti, 2009) was used to remove all outlier values that were more than ‘K’ spreads (K = 4 was used) from the center using the ‘JMP’ statistical software (www.jmp.com). The phenotyping data for all the lines is provided in **Supplementary Table 1**.

### Genotyping

The lines used in this study were genotyped using genotyping-by-sequencing (GBS) described in Poland et al. (2012a). The TASSEL (Trait Analysis by aSSociation Evolution and Linkage) version 5 GBS pipeline (Glaubitz et al., 2014) was used to call marker polymorphisms, aligned to the reference genome (RefSeq v1.0) assembly of the bread wheat variety Chinese Spring (IWGSC, 2018). A total of 6,075,743 GBS tags were aligned to RefSeq v1.0, with an overall alignment rate of 63.98%. A minor allele frequency of 0.01 was used for single nucleotide polymorphisms discovery, and the resulting tags were filtered as described in Juliana et al. (2019), resulting in 78,662 single nucleotide polymorphisms that passed at least one of these filters. These markers were further filtered in each of the populations for missing data less than 60%, minor allele frequency greater than 0.05, and heterozygosity less than 10%, and the lines in each population were also filtered for less than 50% missing data. The final number of lines and markers for the different populations and crop cycles after filtering is given in **Table 1**. Marker imputation was done using the LD-kNNi genotype imputation method (Money et al., 2015) in TASSEL v5 (Bradbury et al., 2007). The genotyping data for all the 23,526 lines is available in https://doi.org/10.6084/m9.figshare.12350000.v1.

**Table 1:** The yield testing stages, cycles, number of lines in each cycle with non-missing data, and the number of filtered markers that were used for predictions.

Yield testing stage	Number of lines	Number of markers
**Stage 1**		
2012–2013	947 lines	6,071 markers
2013–2014	6,408 lines	8,416 markers
2014–2015	7,987 lines	11,982 markers
2015–2016	8,182 lines	11,518 markers
**Stage 2**		
2013–2014	947 lines	6,071 markers
2014–2015	1,012 lines	5,963 markers
2015–2016	1,052 lines	8,402 markers
2016–2017	1,040 lines	8,312 markers
**Stage 3**		
2014–2015	269 lines	6,180 markers
2015–2016	263 lines	5,399 markers
2016–2017	272 lines	6,356 markers
2017–2018	264 lines	5,768 markers

### Statistical Analysis of the Grain Yield Data

The GY BLUEs within each stage, environment, cycle, and breeding population were used to calculate the coefficient of variation, interquartile range, mean, median, range, standard deviation, standard error of the mean and variance.

### Genomic Prediction for Grain Yield in a Subset of Lines Within the Same Site and Year

For genomic prediction of GY in a subset of lines within the same site and year, we used 36 datasets comprising different populations evaluated in different cycles and divided the number of lines in each population into ‘k’-folds for k-fold cross-validations (CV). Cross-validations were performed for the different populations, as follows: (i) **Stage 1 of yield testing**—5,126 to 6,546 lines (fourfolds sampled in 10 independent repetitions) were used as training sets to predict the remaining 1,282 to 1,636 lines (fifthfold); (ii) **Stage 2 of yield testing**—758 to 842 lines (fourfolds sampled in 20 independent repetitions) were used as training sets to predict the remaining 189 to 210 lines (fifthfold); (iii) **Stage 3 of yield testing**—10 to 218 lines (fourfolds sampled in 20 independent repetitions) were used as training sets to predict the remaining 53 to 54 lines (fifthfold). Genomic predictions were done using the genomic-best linear unbiased prediction model (Habier et al., 2013) that was fitted using the ‘R’ package BGLR (Pérez and de los Campos, 2014) and represented as:

(2) $$y=\mu 1+Z_{g}u+\varepsilon$$

where ***y*** represents the GY BLUEs, *μ* is the mean, ***u*** represents the additive genetic effects, and ε is the error term. The joint distribution of the vector of additive genetic effects ***u*** was assumed to be multivariate normal $MN\;\left(0,\;G\sigma_{g}^{2}\right)$where **G** = **ZZ**'/*p* is the genomic relationship matrix calculated using markers (VanRaden, 2008), **Z** is the centered and standardized marker matrix, *p* is the number of markers and $\sigma_{g}^{2}$ is the genetic variance component and the joint distribution of the error (**ε)** was assumed to be $MN\;(0,I\sigma_{e}^{2})$ (**I** is the identity matrix and $\sigma_{e}^{2}$ is the residual variance).

To determine the advantage of using genomic relationships over pedigree-based relationships for predicting GY, we substituted the genomic relationship matrix with the pedigree relationship matrix calculated from the coefficient of parentage. The PAs from these models and all the models used henceforth were calculated as the Pearson’s correlations between the estimated breeding values and the GY BLUEs. We also obtained the full-sib family sizes in all the populations and analyzed the GY range, marker based-relationships and the two-fold cross-validation genomic PAs for 10 big full-sib families comprising 40 to 65 full-sibs. The GY differences and the genomic relationships between the full-sibs were also analyzed.

The relationships between GY predictabilities and the narrow-sense heritabilities were obtained from the Pearson’s correlations between them, where the narrow-sense heritabilities for GY within each population, site, and year were calculated on a line-mean basis across the replicates using the formula:

(3) $$h^{2}=\;\frac{\sigma_{g}^{2}}{\sigma_{g}^{2}+\;\frac{\sigma_{\varepsilon }^{2}}{nreps}}$$

where $\sigma_{g}^{2}$ is the genetic variance calculated using markers, $\sigma_{\varepsilon }^{2}$ is the error variance, and *nreps* is the number of replications. The average information-restricted maximum likelihood algorithm (Gilmour et al., 1995) implemented in the ‘R’ package ‘heritability’ (Kruijer et al., 2015) was used to estimate the genetic and residual variances.

### Genomic Prediction for Minimizing Grain Yield Testing in Stages/Years Within the Selection Site

To determine if GS can be used for minimizing stage(s)/year(s) of GY testing, we used 1,092 lines that were common across Stages 1 and 2 of yield testing and 280 lines that were common across all the three stages of yield testing, resulting in 43 population-stage combinations. The G × E model used was fitted using the BGLR package in ‘R’, and can be represented as:

(4) $$y=\mu 1+Z_{y}\beta_{y}+Z_{g}u_{1}+u_{2}+\varepsilon$$

where ***y*** represent the GY BLUEs; *μ* is the general mean; ***Z*** *_y_* is an incidence matrix for the stage/year, ***β*** *_y_* is the random effect of the year assumed to be multivariate normal $\beta_{y}\sim MN\left(0,\;\sigma_{y}^{2}I\right)$; ***Z*** *_g_* is an incidence matrix that connects the lines with the GY phenotypes; ***u***_1_ represents the random effect of the lines; ***u***_1_ is the G × E interaction, that is assumed to be multivariate normal $u_{2}\sim MN\left(0,\sigma_{gy}^{2}\left(Z_{g}GZ_{g}^{'}\right)\#\left(Z_{y}Z_{y}^{'}\right)\right)$ where # denotes the Hadamard product (cell-by-cell) of the two matrices in parentheses (Jarquín et al., 2014) and **ε** represents the residuals that are also assumed to be multivariate normal and distributed as $\varepsilon \sim MN\left(0,\sigma_{\text{ε}}^{2}I\right)$.The baseline EL model that includes only the main effects of the environment/year and the lines can be represented as:

(5) $$y=\mu 1+\;Z_{y}\beta_{y}+Z_{l}\beta_{l}+\;\varepsilon$$

Where ***y*** represent the GY BLUEs, *μ* is the general mean, ***Z*** *_y_*, ***β*** *_y_* and **ε** represent the same as in Equation 4, ***Z*** *_l_* is an incidence matrix for the lines and ***β*** *_l_* represent the random effect of the lines such that it is multivariate normal $\beta_{l}\sim MN\left(0,\;\sigma_{l}^{2}I\right)$ where $\sigma_{l}^{2}$ represents the variance of the lines.

We also obtained the genetic $\left(\sigma_{g}^{2}\right)$, year $\left(\sigma_{y}^{2}\right)$, genotype x year $\left(\sigma_{gy}^{2}\right)$ and error variance $\left(\sigma_{\text{ε}}^{2}\right)$ components from the G × E model (4) and used them to estimate the narrow-sense heritabilities of GY across stages/years of yield testing in the different environments using the formula below:

6) $$h^{2}=\;\frac{\sigma_{g}^{2}}{\sigma_{g}^{2}+\;\frac{\sigma_{y}^{2}}{nyears}+\;\frac{\sigma_{gy}^{2}}{ngenotypes*nyears}+\;\frac{\sigma_{\varepsilon }^{2}}{ngenotypes*nyears*nreplications}}$$

where *nyears* is the number of years, *ngenotypes* is the number of lines, and *nreplications* is the number of replications. Pearson’s correlations and the genetic correlations between the different stages of yield testing were also estimated. We used the ‘R’ package EMMREML (Akdemir and Okeke, 2015) to obtain the genetic correlations from the genetic covariances across the different stages of yield testing, using the formula (Falconer, 1960),

 (7) $$r_{A}\;=\frac{COV_{XY}}{\sqrt{var_{X}var_{Y}}}$$

where *r_A_* is the genetic correlation between the two stages, *cov_XY_* is the covariance for GY in stages X and Y and *var_X_* and *var_X_* are the variances for GY in stages X and Y. The "emmremlMultivariate" function in EMMREML solves a multivariate Gaussian mixed model that has a known covariance structure and considers the additive genetic (co)variance matrix of GY in different stages, calculated using markers. We also determined the correlations between the PAs from the G × E model and the phenotypic and genetic correlations in addition to the p-values for the significance of the correlations using a two-tailed t-test

### Genomic Prediction for Minimizing Grain Yield Testing in Selection Environments Within a Year

To determine if GS can be used for minimizing GY testing in the managed environments of Obregón within a year, we used the 1,092 lines that were evaluated in six managed Stage 2 environments and 280 lines that were evaluated in three managed Stage 3 environments. The PAs for predicting all other environments from one environment were obtained using the modified G × E model (4) and compared to the modified baseline EL model (5), where the year effects in the models were replaced by the environmental effects for 126 population-environment combinations. The Pearson’s correlations and the genetic correlations (equation 7) between GY BLUEs in these environments were also obtained and compared to the PAs from the G × E model.

## Results

### Grain Yield Data and Statistical Analysis

**Figure 1:**
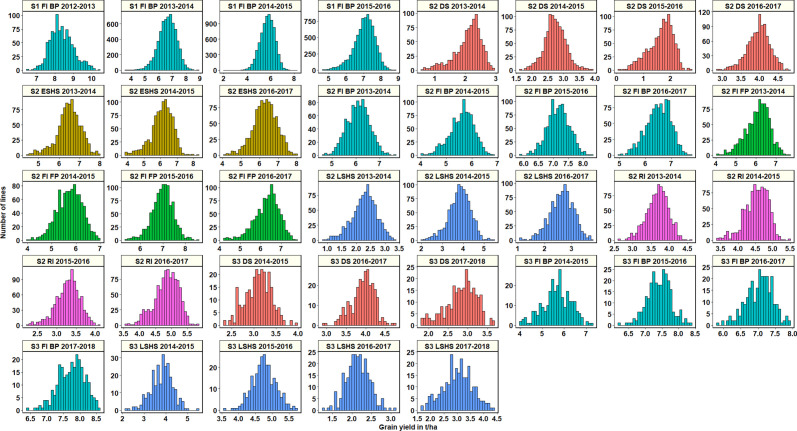
Grain yield distributions for the lines evaluated in the selection-environments of Obregón including Stage 1 full irrigation bed planting (S1 F1 BP), Stage 2 full irrigation bed planting (S2 FI BP), Stage 2 full irrigation flat planting (S2 FI FP), Stage 2 reduced irrigation (S2 RI), Stage 2 drought stress (S2 DS), Stage 2 early-sown heat stress (S2 ESHS), Stage 2 late-sown heat stress (S2 LSHS), Stage 3 full irrigation bed planting (S3 FI BP), Stage 3 drought stress (S3 DS) and Stage 3 late-sown heat stress (S3 LSHS) environment, during the 2012–2013 to 2018–2019 cycles.

Across all datasets, GY was distributed normally (**Figure 1**) and statistical analysis (**Supplementary Table 2**) indicated that in the S1 FI BP environment, the 2015–2016 cycle had the highest mean GY (7.03 ± 0.61 t/ha) followed by cycle 2013–2014 (6.6 ± 0.66 t/ha). Among all the stage 2 yield testing environments, the highest mean GY across cycles was observed in the S2 FI BP environment (6.3 ± 0.65 t/ha), followed by the S2 FI FP environment (6.28 ± 0.53 t/ha) and the S2 ESHS environment (6.28 ± 0.19 t/ha). The lowest mean GY was observed in the S2 DS environment (2.6 ± 1 t/ha), followed by the S2 LSHS environment (2.9 ± 0.8 t/ha) and the S2 RI environment (4.08 ± 0.72 t/ha).

In the S2 FI BP and S2 FI FP environments, the highest mean GY were observed in the 2015–2016 cycle (7.15 ± 0.37 t/ha and 7 ± 0.47 t/ha, respectively). In the S2 ESHS and S2 LSHS environments, the highest mean GY were observed in the 2013–2014 cycle (6.47 ± 0.6 t/ha) and in the 2014–2015 cycle (3.8 ± 0.53 t/ha), respectively. In the S2 RI and S2 DS environments, we observed the highest mean GY in the 2016–2017 cycle (4.8 ± 0.38 and 3.9 ± 0.3 t/ha, respectively).

Among all the stage 3 yield testing environments, the highest mean GY across cycles was observed in the S3 FI BP environment (7 ± 0.9 t/ha), followed by the S3 LSHS environment (3.4 ± 1.1 t/ha) and the S3 DS environment (3.3 ± 0.6 t/ha). In the S3 FI BP, S3 LSHS and the S3 DS environments, the highest mean GY were in the 2017–2018 cycle (7.7 ± 0.4 t/ha), 2015–2016 cycle (4.8 ± 0.35 t/ha), and the 2016–2017 cycles (3.9 ± 0.3 t/ha), respectively.

### Genomic Prediction for Grain Yield in a Subset of Lines Within the Same Site and Year

#### Comparison of Genomic and Pedigree Prediction Accuracies

**Figure 2:**
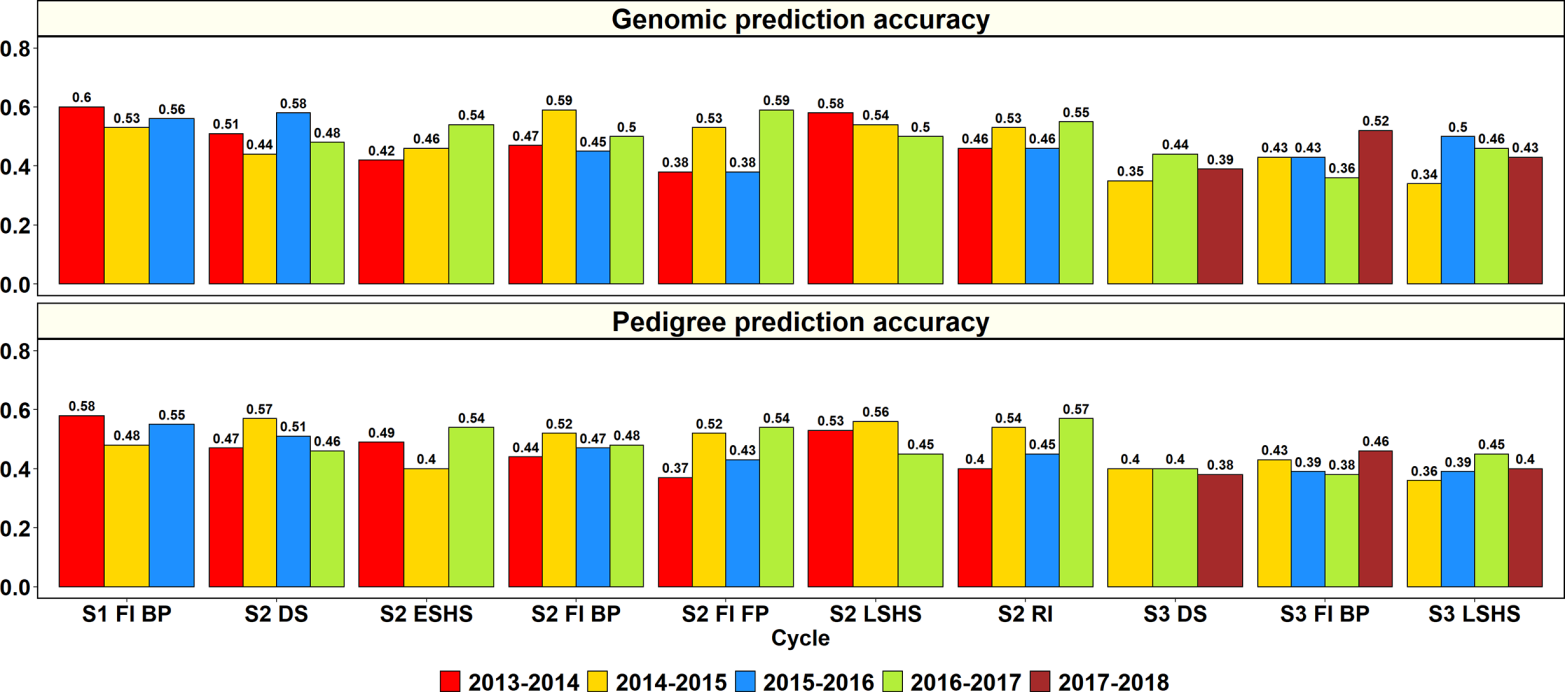
Genomic and pedigree-based prediction accuracies for grain yield within years and sites in the selection-environments of Obregón: Stage 1 Full irrigation bed planting environment (S1 F1 BP), Stage 2 Full irrigation bed planting (S2 FI BP), Stage 2 Full irrigation flat planting (S2 FI FP), Stage 2 Reduced irrigation bed planting (S2 RI), Stage 2 Drought stress flat planting (S2 DS), Stage 2 Early-sown heat stress bed planting, (S2 ESHS), Stage 2 Late-sown heat stress bed planting (S2 LSHS), Stage 3 Full irrigation bed planting (S3 FI BP), Stage 3 Drought stress flat planting (S3 DS), and Stage 3 Late-sown heat stress bed planting (S3 LSHS). The accuracies were obtained from fivefold cross-validations across different cycles as follows: (i) S1 of yield testing at Obregón—1,282 to 1,636 lines were predicted from training sets of 5,126 to 6,546 lines (2013–2014 to 2015–2016 cycles); (ii) S2 of yield testing at Obregón—189 to 210 lines were predicted from training sets of 758 to 842 lines (2013–2014 to 2016–2017 cycles); (iii) Stage 3 of yield testing at Obregón—53 to 54 lines were predicted from training sets of 210 to 218 lines (2014–2015 to 2017–2018 cycles). There were no significant differences between genomic and pedigree-based prediction accuracies.

The GY PAs in a subset of lines within the same site and year using genomic and pedigree relationships were obtained (**Figure 2**). In S1 of yield testing at Obregón, where 1,282 to 1,636 lines were predicted from training sets of 5,126 to 6,546 lines across three cycles, the mean genomic and pedigree-based PAs were 0.56 ± 0.04 and 0.54 ± 0.05, respectively. The S1 FI BP 2013–2014 cycle had the highest genomic and pedigree PAs (0.6 and 0.58, respectively) and the differences between the genomic and pedigree PAs in the different cycles ranged from 0.01 to 0.05.

In S2 of yield testing, where 189 to 210 lines were predicted from training sets of 758 to 842 lines, the mean genomic and pedigree PAs across the six environments were 0.5 ± 0.06 and 0.49 ± 0.06, respectively. The mean genomic PA across cycles was highest in the S2 LSHS environment (0.54), followed by the S2 DS (0.5), S2 FI BP (0.5), S2 RI (0.5), S2 ESHS (0.47), and S2 FI FP (0.47) environments. Similarly, the mean pedigree PA was highest in the S2 LSHS environment (0.51), followed by the S2 DS (0.5), S2 RI (0.4), S2 FI BP (0.48), S2 ESHS (0.48), and S2 FI FP (0.47) environments.

In the S2 LSHS, S2 FI BP, and S2 DS environments, the highest genomic PAs were observed in the 2013–2014 cycle (0.58), 2014–2015 cycle (0.59), and the 2015–2016 cycle (0.58), respectively. In the S2 ESHS, S2 FI FP, and S2 RI environments, the highest genomic PAs were observed in the 2016–2017 cycle and were 0.54, 0.59 and 0.57, respectively. Across all the 22 S2 datasets, the genomic PAs were similar to the pedigree PAs or provided increases ranging between 0.01 and 0.07 in 15 datasets and were 0.01 to 0.13 lower than the pedigree PAs in the remaining seven datasets.

In S3, where 53 to 54 lines were predicted from training sets of 210 to 218 lines, the mean genomic and pedigree PAs across the three environments were 0.42 ± 0.06 and 0.4 ± 0.03, respectively, with the highest mean PAs in the S3 FI BP environment. The mean genomic and pedigree PAs across cycles were highest in the S3 FI BP (0.44 and 0.42, respectively) environment, followed by the S3 LSHS (0.43 and 0.4, respectively) and S3 DS (0.39) environments.

The highest genomic PAs in the S3 DS, S3 FI BP, and S3 LSHS environments were observed in the 2016–2017 cycle (0.44), 2017–2018 cycle (0.52), and in the 2015–2016 cycle (0.5), respectively. Across all the 11 S3 datasets, the genomic PAs were similar to the pedigree PAs or provided increases ranging between 0.01 and 0.11 in eight datasets and were 0.02 to 0.05 lower than the pedigree PAs in the remaining three datasets.

**Figure 3:**
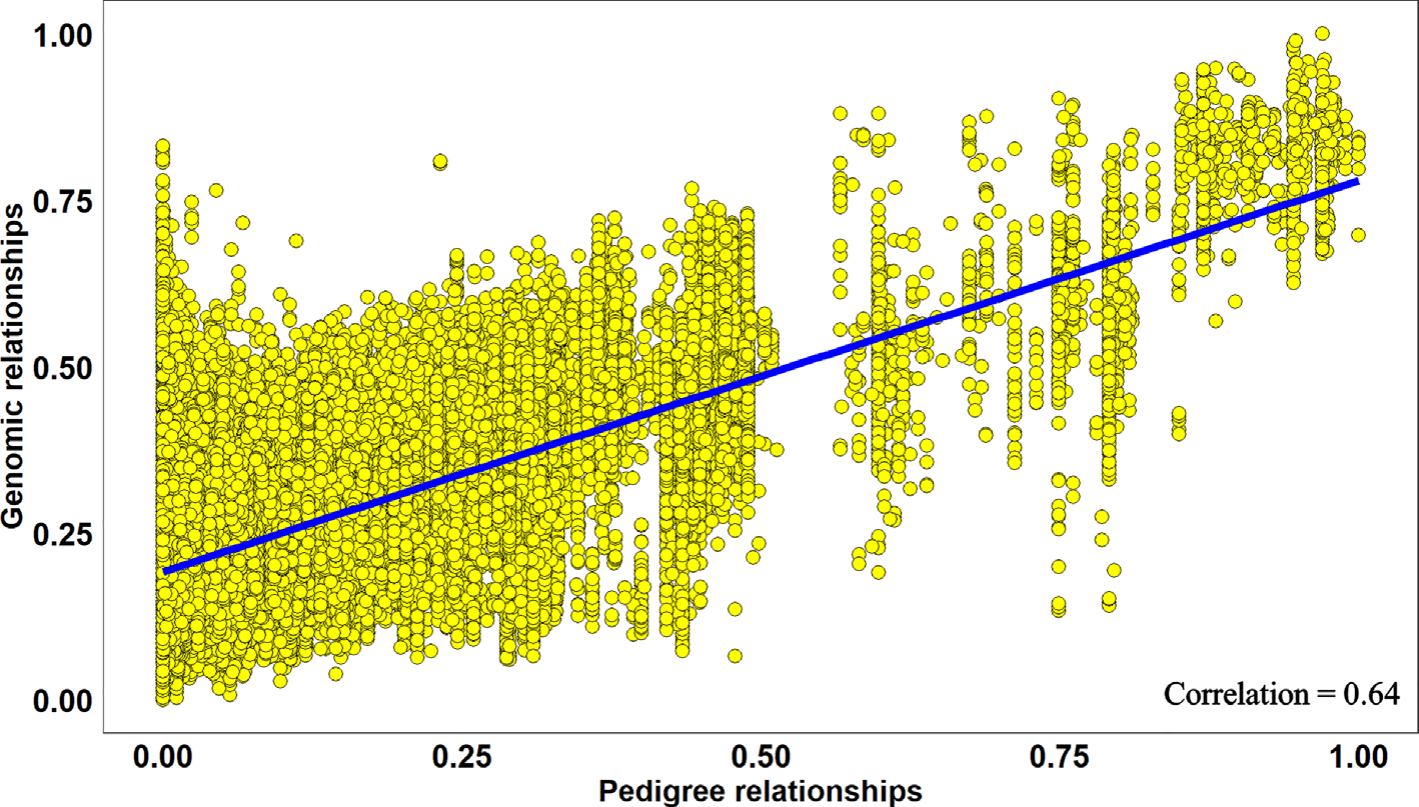
Scatter plot of rescaled (between 0 and 1) genomic relationships and pedigree relationships for 1,015 lines in the Stage 2 2016–2017 panel, fitted with a linear regression line. The correlation between the genomic and pedigree relationships was high (0.64), but several discrepancies *i.e.* high genomic and low pedigree relationships and *vice versa* were observed.

Overall, across all the stages and environments of yield testing, we observed that the genomic PAs across all the datasets were similar to the baseline pedigree Pas, and the p-value for the test of significance of the differences between them (0.07) was not significant at the 0.001 level. However, when the pairwise genomic relationships between lines in a panel (S2 16–17) were plotted against their pedigree relationships, we observed several discrepancies between the relationships (high genomic and low pedigree relationships and vice versa) despite a high correlation (0.64) (**Figure 3**), indicating that the genomic and pedigree relationships are dissimilar in many lines.

#### Genomic Prediction Accuracies in Full-Sib Families

**Figure 4:**
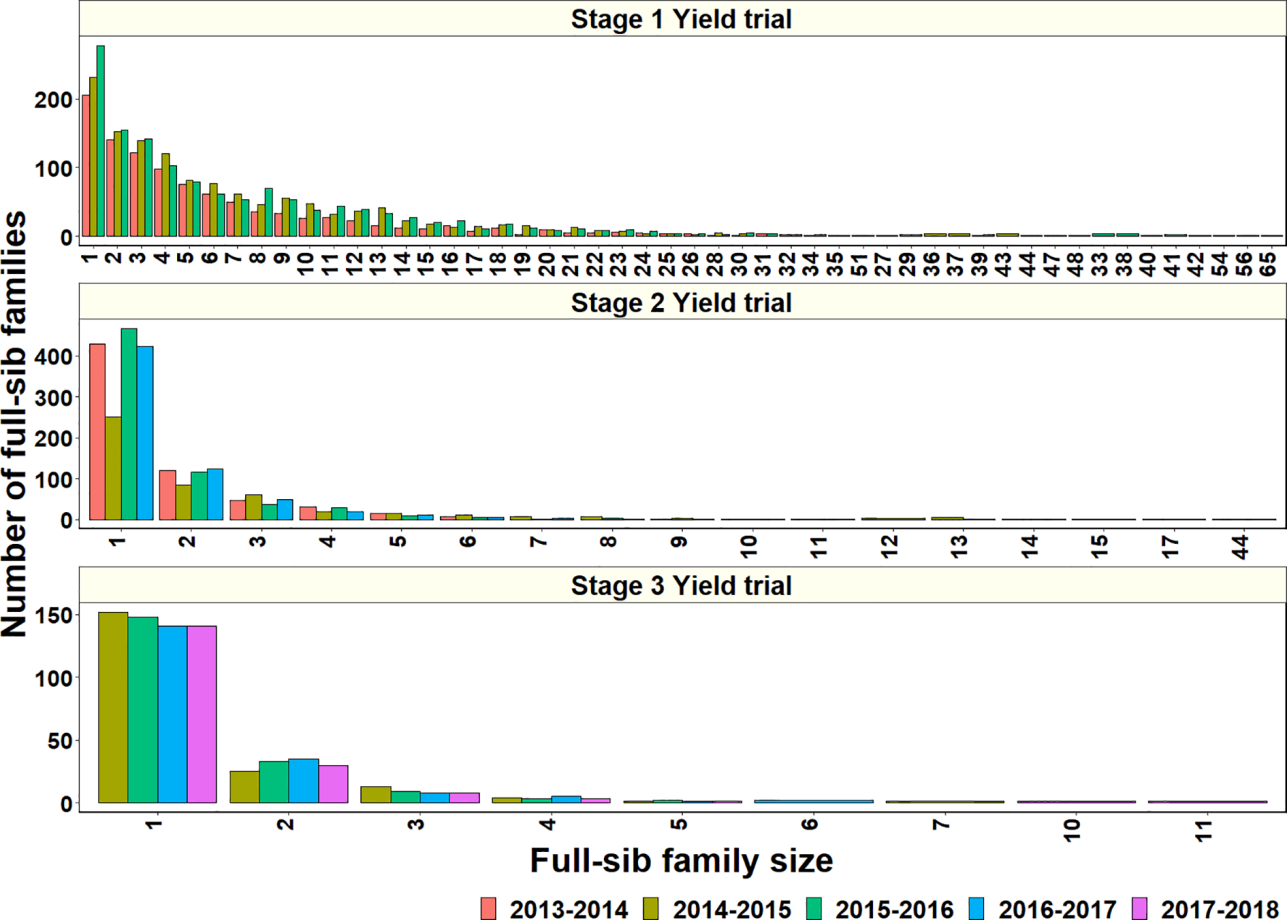
Full-sib family sizes and the number of full-sib families in Stage 1 Yield trials (2013–2014 to 2015–2016 cycles), Stage 2 Yield trials (2013–2014 to 2016–2017 cycles), and Stage 3 Yield trials (2014–2015 to 2017–2018 cycles).

To explore why genomic relationships had no advantage over pedigree relationships, the full-sib family sizes in the populations were analyzed. The largest full-sib family sizes in S1, S2, and S3 of yield testing were 65, 44, and 11, respectively (**Figure 4**). Furthermore, to understand the genomic and phenotypic variation among the full-sib progenies, the genomic relationships, GY range and PAs in the biggest full-sib families with 40 to 64 full-sibs and good genotyping data were analyzed (**Supplementary Table 3**). The twofold CV genomic PAs ranged between 0.05 and 0.52, and GY differences ranged between 1.55 t/ha and 2.58 t/ha in the full-sib families. The DANPHE #1*2/CHYAK//MUTUS*2/HARIL #1 family with 41 full-sibs was the least predicted (mean PA of 0.05) and the SAUAL/MUTUS/4/KACHU #1//WBLL1*2/KUKUNA/3/BRBT1*2/KIRITATI family with 45 full-sibs was the best predicted (mean PA of 0.52).

**Figure 5:**
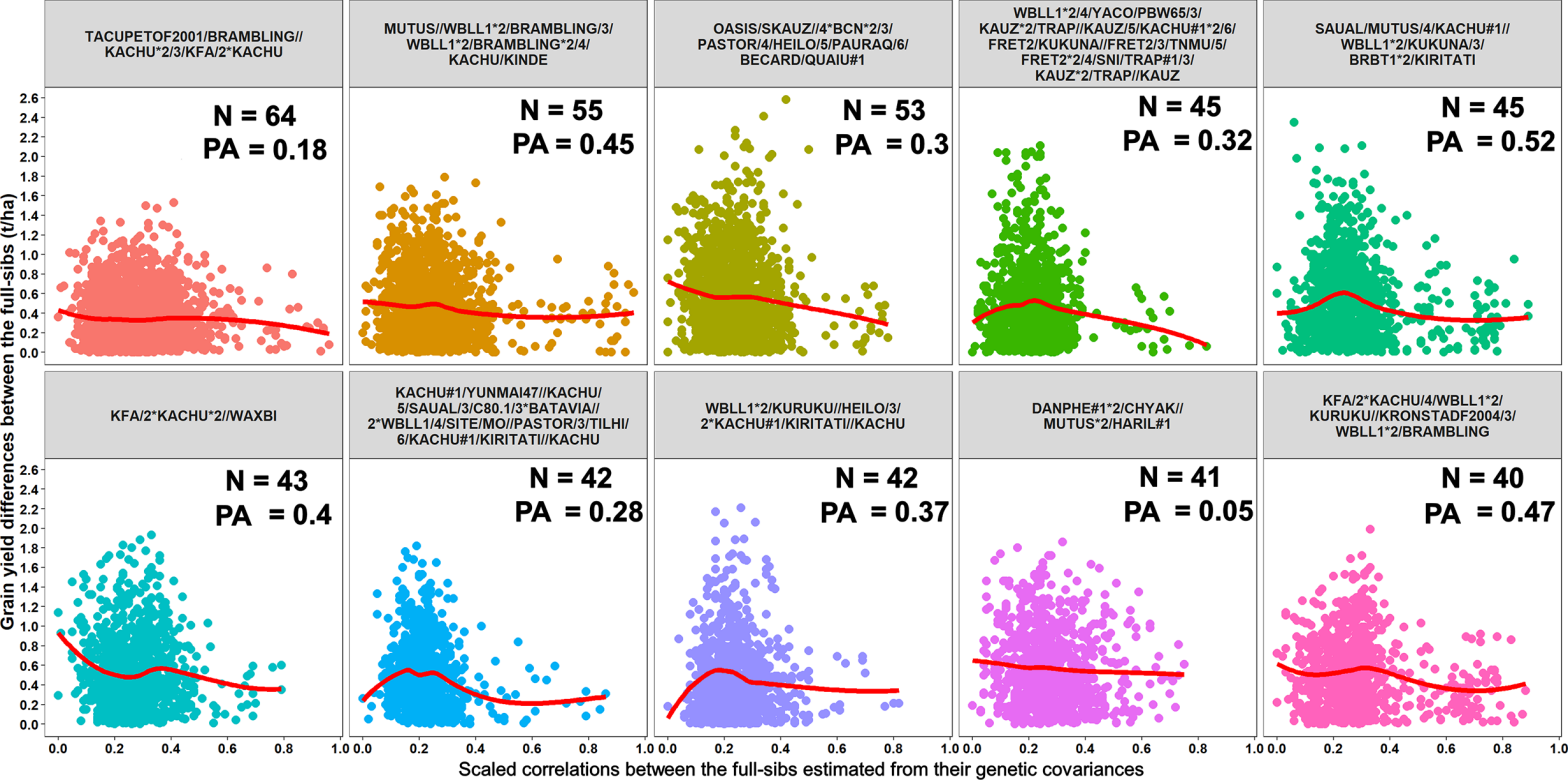
Scatter plots showing the differences in grain yield between the full-sibs and the scaled correlations between the full-sibs estimated from their genetic covariances in 10 full-sib families. The number of full-sibs in each of these families (N) and the twofold cross-validation genomic prediction accuracies (PAs) are shown. The relationships between the full-sibs did not have a linear relationship with the grain yield differences between the full-sibs *i.e.* closely related full-sibs did not have small differences in grain yield and *vice-versa*. The full-sibs with relationships between 0.1 and 0.4 had a high range in their grain yield differences.

**Figure 6:**
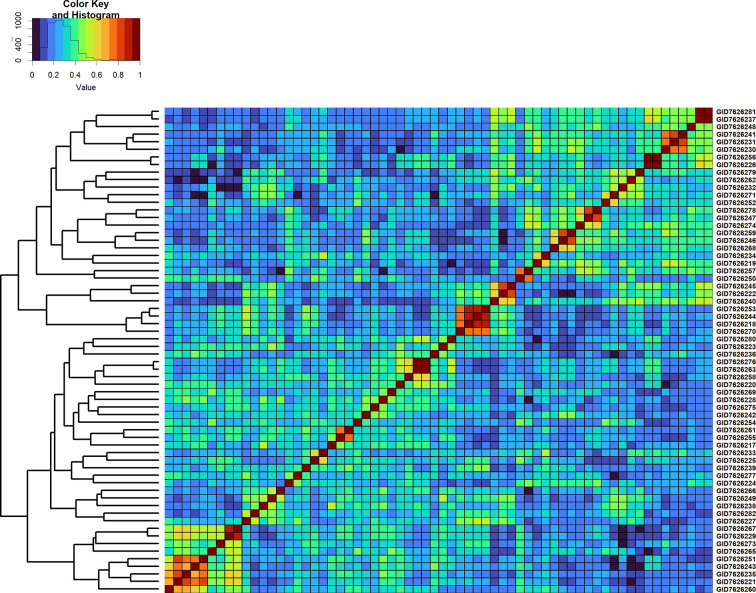
Genomic relationship matrix scaled between 0 and 1 and clustering of 64 full-sibs from the cross, TACUPETOF2001/BRAMBLING//KACHU*2/3/KFA/2*KACHU (Cross ID 590394).

**Figure 7:**
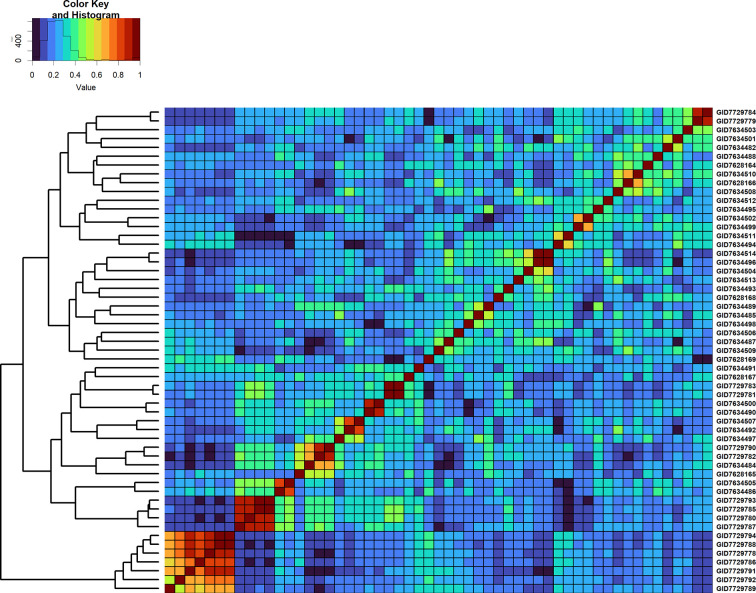
Genomic relationship matrix scaled between 0 and 1 and clustering of 55 full-sibs from the cross, MUTUS//WBLL1*2/BRAMBLING/3/WBLL1*2/BRAMBLING*2/4/KACHU/KINDE (Cross ID 590762).

Furthermore, we compared GY differences between the full-sibs with their genomic relationships and observed that the relationships between full-sibs had no linear relationships with the GY differences between full-sibs in the different families (**Figure 5**). The full-sibs with relationships between 0.1 and 0.4 had a high range in their GY differences. We also observed a clear deviation from the expected relationship of 0.5 between full-sibs indicating that the markers captured the Mendelian sampling variation among full-sibs (**Figures 6**, **7**). While a large number of full-sibs in all the families had relationships between 0.2 and 0.4, several full-sibs had low relationships with each other in the different families, indicating that all full-sibs were not highly related.

#### Relationship Between Genomic Prediction Accuracies and Heritabilities

To understand the effect of GY heritabilities on the PAs, the narrow-sense line-mean heritabilities and their relationships with the PAs were analyzed (**Supplementary Table 4**). While the mean heritabilities in the different environments and years ranged between 0.63 and 0.89, the highest mean heritability across years was observed in the S2 LSHS environment (0.89), followed by the S2 DS environment (0.87) and the S2 ESHS environment (0.83). Across all the datasets, the PAs had a low correlation of 0.24 with the heritabilities, the PAs were always lower than the heritabilities, and the differences between them ranged from 0.04 to 0.50.

### Genomic Prediction for Minimizing Grain Yield Testing in Stages/Years Within the Selection Site

The potential of GS for minimizing GY testing across stages/years in the SEs of Obregón was assessed using lines that were evaluated in more than one stage, across four cohorts that represented a set of lines evaluated in different stages or cycles (**Figure 6**, **Supplementary Table 5**). Genomic predictions from the S1 FI BP environment to the six environments of S2 using the G × E model resulted in mean PAs ranging between 0.08 and 0.39 across the four cycles. The Stage 2 environments that were best predicted from the S1 FI BP environment and had high mean PAs included the S2 FI BP (0.39), S2 ESHS (0.38), and S2 FI FP (0.30) environments. We also observed that the S1 FI BP environment had low predictive abilities for the following environments as observed by the mean PAs: S2 DS (0.08), S2 LSHS (0.17), and S2 RI (0.21).

When the three environments in S3 were predicted from the S1 FI BP environment, the mean PAs ranged between 0.01 and 0.39, and when they were predicted from the same environments in S2, the mean PAs were higher and ranged between 0.31 and 0.51. The S1 FI BP environment had the highest mean PA (0.39) for the S3 FI BP environment and the lowest mean PA (0.01) for the S3 DS environment. Considering the mean PAs of the Stage 3 environments from their corresponding Stage 2 environments, the S3 FI BP environment was the best predicted (0.51), followed by the S3 LSHS environment (0.36) and the S3 DS environment (0.31). Overall, across all the datasets, the highest mean PA was observed when S3 FI BP was predicted from S2 FI BP. On comparing the PAs from the G × E model to the baseline EL model, we observed no significant differences, and the p-value for the test of significance of the differences between them (0.01) was not significant at the 0.001 level (**Supplementary Table 5**).

**Figure 8:**
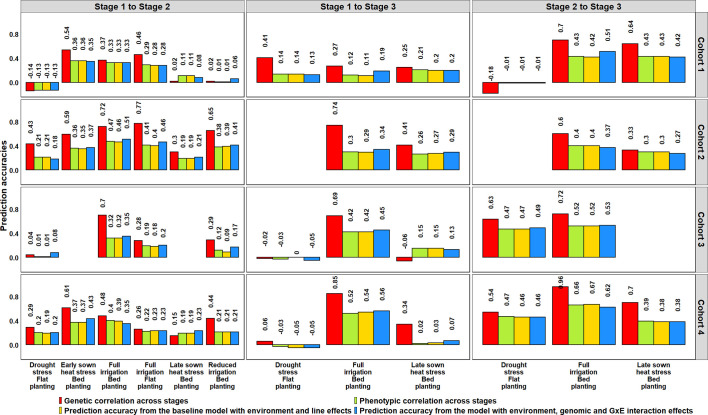
Genetic and phenotypic correlations across stages of yield testing in Obregón and prediction accuracies from the baseline model with environment and line effects and the model with environment, genomic and genotype × environment (G × E) interaction effects. About 1,092 lines were common across Stages 1 and 2, and 280 lines were common across all the stages of yield testing. The cohorts represent a set of lines that were evaluated during three crop cycles: Cohort 1 represents lines evaluated during 2012–2013 (Stage 1), 2013–2014 (Stage 2), and 2014–2015 (Stage 3) cycles; Cohort 2 represents lines evaluated during 2013–2014 (Stage 1), 2014–2015 (Stage 2), and 2015–2016 (Stage 3) cycles; Cohort 3 represents lines evaluated during 2014–2015 (Stage 1), 2015–2016 (Stage 2), and 2016–2017 (Stage 3) cycles; and Cohort 4 represents lines evaluated during 2015–2016 (Stage 1), 2016–2017 (Stage 2), and 2017–2018 (Stage 3) cycles. There were no significant differences between the phenotypic correlations and the prediction accuracies from the baseline model and the G × E model across the stages.

We also determined the phenotypic and genetic correlations between different stages of yield testing and observed that the PAs from the G × E model were similar to the phenotypic correlations (p-value for the test of significance of the differences between them was 0.02) compared to the genetic correlations (**Figure 8**). However, the genetic correlations had strong correlations with the phenotypic correlations (0.91) and were on average 0.18 higher than the phenotypic correlations. We also observed significant correlations between the environments in different stages of yield testing (at a p-value threshold of 0.001), except between the S1 FI BP environment and the S3 DS and S3 LSHS environments (**Supplementary Table 5**).

To understand why the G × E model had no advantage over the baseline EL model, we partitioned the phenotypic variance into the genetic, year, genotype × year and error variance components (**Table 2**). While the G × E variances were only 0.4 to 1.2 times the genetic variances across all the stages, the year variances were 3.2 to 11.3 times the genetic variances, making them the largest contributors to the variation occurring across years of testing. Furthermore, narrow-sense heritabilities across stages/years were estimated from the variance components, and they ranged from 0.11 to 0.68.

**Table 2:** Marker-based estimates of genetic (*σ* ^2^ *_g_*), year (*σ* ^2^ *_y_*), genotype × year (*σ* ^2^ *_g_*_× y_) and error (*σ* ^2^ *_ε_*) variance components, variance ratios, and narrow-sense heritabilities across stages of yield testing in Obregón.

Variance components, variance ratios, and heritabilities	Cohort	Full irrigation Bed planting Stage 1 and Stage 2	Full irrigation Bed planting Stage 1 and Stage 3	Full irrigation Bed planting Stage 2 and Stage 3	Late sown heat stress Stage 2 and Stage 3	Drought stress Stage 2 and Stage 3
*σ* ^2^ *_g_*	1	0.11	0.12	0.11	0.11	0.02
*σ* ^2^ *_y_*		1.30	2.05	0.30	0.56	0.26
*σ* ^2^ *_g_*_× y_		0.08	0.16	0.05	0.05	0.06
σ^2^_ε_		0.18	0.22	0.15	0.09	0.06
*σ* ^2^ *_g_*: *σ* ^2^ *_y_*: *σ* ^2^ *_g_*_×_ *_y_*: σ^2^_ε_		1:11.7:0.7:1.6	1:16.4:1.3:1.8	1:2.8:0.5:1.4	1:4.9:0.5:0.8	1:10.7:2.6:2.3
*h^2^*		0.15	0.11	0.41	0.29	0.16
*σ* ^2^ *_g_*	2	0.12	0.05	0.07	0.06	–
*σ* ^2^ *_y_*		0.82	0.10	0.72	0.20	–
*σ* ^2^ *_g_*_×_ *_y_*		0.05	0.03	0.05	0.06	–
*σ* ^2^ *_ε_*		0.08	0.07	0.05	0.06	–
*σ* ^2^ *_g_*: *σ* ^2^ *_y_*: *σ* ^2^ *_g_*_×_ *_y_*: *σ* ^2^ *_ε_*		1:6.6:0.4:0.7	1:1.9:0.6:1.4	1:9.8:0.6:0.7	1:3.2:0.9:1	–
*h^2^*		0.23	0.51	0.17	0.38	–
*σ* ^2^ *_g_*	3	0.06	0.08	0.10	–	0.08
*σ* ^2^ *_y_*		0.32	0.17	0.09	–	1.27
*σ* ^2^ *_g_*_×_ *_y_*		0.03	0.02	0.02	–	0.05
*σ* ^2^ *_ε_*		0.06	0.06	0.05	–	0.06
*σ* ^2^ *_g_*: *σ* ^2^ *_y_*: *σ* ^2^ *_g_*_×_ *_y_*: *σ* ^2^ *_ε_*		1:5.6:0.4:1.1	1:2.2:0.3:0.8	1:0.9:0.2:0.5	–	1:16.6:0.6:0.8
*h^2^*		0.26	0.47	0.68	–	0.11
*σ* ^2^ *_g_*	4	0.07	0.08	0.15	0.10	0.07
*σ* ^2^ *_y_*		0.42	0.08	0.32	0.14	0.46
*σ* ^2^ *_g_*_×_ *_y_*		0.04	0.02	0.02	0.06	0.04
*σ* ^2^ *_ε_*		0.08	0.06	0.05	0.09	0.05
*σ* ^2^ *_g_*: *σ* ^2^ *_y_*: *σ* ^2^ *_g_*_×_ *_y_*: *σ* ^2^ *_ε_*		1:6.2:0.6:1.1	1:1:0.2:0.7	1:2.1:0.2:0.3	1:1.4:0.7:0.9	1:6.6:0.5:0.7
*h^2^*		0.24	0.66	0.49	0.58	0.23
Mean *h^2^*	All	0.22 ± 0.05	0.44 ± 0.23	0.44 ± 0.21	0.42 ± 0.15	0.17 ± 0.06
Mean *σ* ^2^ *_g_*: *σ* ^2^ *_y_*: *σ* ^2^ *_g_*_×_ *_y_*: *σ* ^2^ *_ε_*		1:7.5:0.5:1.1	1:5.4:0.6:1.2	1:3.9:0.4:0.7	1:3.2:0.7:0.9	1:11.3:1.2:1.3

### Genomic Prediction for Minimizing Grain Yield Testing in Selection Environments Within a Year

**Figure 9:**
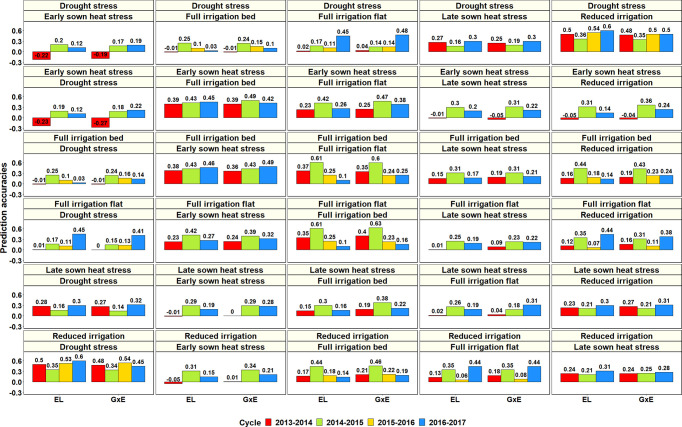
Prediction accuracies across the Stage 2 yield testing environments from the baseline model with environment and line effects (EL model) and the model with environment, genomic and genotype × environment (G × E) interaction effects (G × E model) for about 1,092 lines in four cycles. There were no significant differences between the prediction accuracies from the baseline model and the G × E model.

To determine if GS can be used for minimizing GY testing in the managed SEs of Obregón, the PAs across 126 SE pairs in different cycles were obtained using the G × E model and compared to a baseline EL model (**Figure 9**, **Supplementary Table 6**). Overall, we observed a negligible mean increase (0.02 ± 0.05) using the G × E model over the baseline EL model, but there were significant differences between the pairwise PAs (p-value for the test of significance of the differences between them was 0.001).

Considering only the G × E PAs within the Stage 2 environments: the S2 DS environment had the highest mean PA for the S2 RI environment (0.46 ± 0.07), the S2 ESHS environment had the highest mean PA for the S2 FI BP (0.43 ± 0.05) and the S2 FI FP (0.37 ± 0.1) environments, the S2 FI BP environment had the highest mean PA for the S2 ESHS (0.43 ± 0.07) and the S2 FI FP (0.36 ± 0.17) environments, the S2 FI FP environment had the highest mean PA for the S2 FI BP (0.36 ± 0.21) and the S2 ESHS (0.32 ± 0.08) environments, the S2 LSHS environment had the highest mean PA for the S2 FI BP (0.26 ± 0.1) and the S2 RI BP (0.26 ± 0.05) environments and the S2 RI environment had the highest mean PA for the S2 DS (0.45 ± 0.08) environment. Overall, the Stage 2 environment-pairs, RI and DS and FI BP, FI FP and ESHS were best predicted from each other. Within the Stage 3 environments, the mean PAs from the G × E model across the three environments were low (ranged from 0.06 to 0.23) and the best predicted Stage 3 environment pair was S3 FI BP and S3 LSHS.

We also analyzed the phenotypic and genetic correlations between the different environments (**Supplementary Table 6**) and observed that they had a strong relationship with each other (correlation of 0.91) and also with the PAs from the G × E model (correlation of 0.95 and 0.89, respectively). While the phenotypic correlations between the different environments ranged from −0.26 to 0.63, the genetic correlations ranged from −0.55 to 0.89 and were on average 0.10 ± 0.13 higher than the phenotypic correlations. Considering the p-values for the significance of both phenotypic and genetic correlations, most environments were significantly correlated with another in at least two years (at a p-value threshold of 0.001), except for the S3 LSHS and S3 DS environments. We also observed that the phenotypic correlations between environments explained 90.8% variation in the PAs from the G × E model and had a highly significant association with them (p-value of 3.1E-66), while the genetic correlations explained only 79.1% of the variation in PAs and were also significant (p-value of 6.2E-44).

## Discussion

In this study, we draw inferences from predictive modeling of GY using 420 different populations, environments, cycles, and model combinations that will have major implications on the implementation of GS for GY in wheat breeding programs. We evaluated the potential of genomic predictions for minimizing the number of replications and lines tested within a site and year and obtained mean PAs of 0.56, 0.5 and 0.42 in Stages 1, 2, and 3 of yield testing respectively, with slight decreases in PAs in the advanced yield trial nurseries that were characterized by fewer and highly selected lines with a narrow GY range. These PAs are within the range or slightly higher than those obtained in previous CV studies for wheat GY: 0.32 (Poland et al., 2012b), 0.22 (Heffner et al., 2011), 0.46–0.63 (Howard et al., 2019), 0.37–0.51 (Juliana et al., 2018b), and 0.44–0.57 (Juliana et al., 2018a).

We also observed that the mean pedigree PAs in Stages 1, 2, and 3 (0.54, 0.49, and 0.4, respectively) were similar to the genomic PAs, clearly indicating that genomic predictions offer no advantage over pedigree predictions for minimizing the number of replications and lines tested within a site and year in all the three yield testing stages at CIMMYT despite speculations of a high gain from GS in the preliminary yield trial stage (Endelman et al., 2014; Gaynor et al., 2017). The similarity in PAs from genomic and pedigree relationships can be attributed to the small full-sib family sizes in the yield testing stages, and several previous studies have reported comparable PAs using genomic and pedigree relationships, and only marginal increases in PAs using genomic relationships in advanced breeding lines (Crossa et al., 2010; Juliana et al., 2017; Juliana et al., 2018b; Howard et al., 2019). However, we have also reported only a moderate correlation of 0.64 between the genomic and pedigree relationships and a deviation from the expected relationship of 0.5 between the full-sibs, indicating that there are differences between the genomic and pedigree relationships, part of which can be attributed to pedigree and genotyping errors and the markers not capturing rare allelic differences between lines. While some studies have reported a marginal increase in PAs using combined genomic and pedigree relationships (Crossa et al., 2010; Juliana et al., 2017; Juliana et al., 2018b; Howard et al., 2019), the low value added by genomic relationships in this study suggests that wheat breeding programs with a well-maintained pedigree and family structures like that of CIMMYT in the yield testing stages can just use the available inexpensive pedigree relationship based predictions to minimize the lines in these stages. In addition, breeding programs that have a large number of replications and PAs comparable to the within environment heritabilities can substitute some of the replications with pedigree-based predictions.

We also report the genomic relationships and GY differences between 11,084 full-sib pairs from ten full-sib families and their GY predictabilities that provide excellent insights into the Mendelian sampling variation and predictabilities in full-sib families. We demonstrate interesting cases where the markers predicted the Mendelian sampling variation between the full-sibs well, but the relationships between the full-sibs were low (even after the full-sibs had undergone several cycles of selection and had some degree of phenotypic similarities) and resulted in low to moderate PAs (0.05 to 0.52). While the narrow GY variation in these full-sibs due to selection and small training populations could have also resulted in low predictabilities, the following question warrants further research: What is the optimum family-size and GY range that will give genomic relationships advantage over the pedigree relationships in the yield testing stages and would it benefit breeding programs to increase family-sizes in these generations at the cost of genetic diversity?

While yield trial nurseries can be restructured to have large family sizes and harness the potential of GS, it should be noted that breeders generally select progenies from multiple crosses in advanced generations to maintain genetic diversity instead of co-selecting full-sibs, which might not favor large family sizes. In addition, not all crosses result in large families with good individuals, due to selection for multiple traits including disease resistance, phenology, plant height *etc.* in earlier generations. Hence, an alternative strategy could be to explore the value of GS for GY in early-generations where large family sizes are expected to provide genomic relationships advantage over pedigree relationships. However, this needs careful consideration of the following questions: (i) Will there be cost-benefits and increased genetic gains by implementing GS in early-generations, considering the huge cost of genotyping and the need to develop infrastructure for genotyping thousands of early-generation progenies? (ii) What is the reliability and stability of early-generation GY predictions in populations, given that the heritability of GY in these generations is low and the progenies are segregating?

We explored the possibility of using genomic predictions with the G × E model to minimize years of GY testing and observed inconsistent PAs across years with a considerable range when the following similar environments were predicted across years: S2 FI BP from S1 FI BP—0.33 to 0.51, S3 FI BP from S1 FI BP—0.19 to 0.56, S3 FI BP from S2 FI BP—0.37 to 0.62, S3 DS from S2 DS—−0.01 to 0.49 and S3 LSHS from S2 LSHS—0.27 to 0.42. In addition, we observed no advantage of the G × E model over the baseline EL model for the scenarios analyzed despite several studies reporting some increase in PAs by modeling G × E in other scenarios (Burgueño et al., 2012; Heslot et al., 2013; Lopez-Cruz et al., 2015; Cuevas et al., 2016; Jarquín et al., 2017). This can be attributed to the large year variance components and the small G × E variance components in the datasets analyzed in this study, clearly indicating that G × E models are not always useful and GY prediction in different years is challenging due to an amalgam of fluctuating factors like edaphic, nutrient, climatic, biotic stresses and management conditions of environments that are not known or predictable beforehand (Allard and Bradshaw, 1964; Goodchild and Boyd, 1975; Hill, 1975; Bell and Fischer, 1994; Dawson et al., 2013; Storlie and Charmet, 2013). In addition, the low to moderate phenotypic correlations observed between GY evaluated in different years (0.41, 0.37, and 0.31, respectively in the FI BP, DS and LSHS environments) indicate the dissimilar natural environment of Obregón in different years. While this is conducive for assessing the temporal stability of lines and rigorous testing under diverse environments probably drives the successful wide adaptation and yield stability of CIMMYT lines in different geographical regions, it is also an unideal scenario for implementing GS that relies on the similarity between environments for good predictions. However, these results are specific to CIMMYT’s yield testing in Obregón, and further studies on across-year GY prediction in other locations are needed.

We also evaluated the use of genomic predictions for minimizing the number of environments evaluated in Stages 2 and 3 and observed very low PAs across the Stage 3 environments (average of 0.14 ± 0.14 and ranged from −0.13 to 0.39), indicating that the three Stage 3 environments were different and cannot be minimized using genomic predictions. In Stage 2, the FI BP, FI FP and ESHS environments had a moderate predictability among them (mean PA of 0.37 ± 0.12), as well as the RI and DS environments (mean PA of 0.45 ± 0.07). However, across years, we observed inconsistencies and a large range in the PAs of the FI BP, FI FP, and ESHS environments (0.16 to 0.63) and a moderate range in the PAs of the RI and DS environments (0.34 to 0.54), indicating that the predictabilities of these environments are not constant across years to reliably use genomic predictions and minimize phenotyping in one or more of them. We also observed that the G × E model provided no advantage over the baseline EL model in predicting across environments within a year, similar to the scenario of predictions across years, suggesting that the effect of the G × E interactions across environments are not as large as the effects of the environments themselves to provide a significant boost in PAs.

Overall, our results indicate that using genomic predictions and G × E models to minimize years and environments of yield testing is not an ideal solution to improving the GY and climate-resilience of wheat as genomic predictions entirely rely on the evaluated GY for training models and predictions in a different environment requires mandatory evaluation of some related genotypes in that environment. Furthermore, the risks associated with minimal yield testing, and substitution of multi-environment testing with inconsistent genomic predictions should be recognized as the unstable lines that result from these will be fraught with risks to farmer’s livelihoods and food security. Since the goal of plant breeding is to develop varieties that are well-buffered to withstand unpredictable environment fluctuations (Allard and Bradshaw, 1964), and stability is the key component in farmer’s adoption decisions (Barah et al., 1981; Singh et al., 2007), multi-environment testing (Fox et al., 1985) is indispensable for sustaining wheat productivity in accordance with the safety-first approach (Eskridge, 1990). In addition, most smallholder farmers in developing countries grow wheat on less than 2 ha, where the best risk-mitigating measure is to develop varieties that can cope with major year-to-year environmental changes, and breeding for such lines with climate-resilience and wide-adaptability requires replicated evaluation over time and space (Hurd, 1969; Ghaderi et al., 1980).

In conclusion, our results provide excellent insights into the predictability of a quantitative trait like GY. The prediction scenarios and the results presented in this study will enable wheat breeding programs to determine the appropriate stages for evaluating GS, understand the complexities in predicting GY, understand the value of using GS and thereby have important implications on the future design of GY evaluation and prediction strategies. While this study does not discount the application of genomics for GY in wheat, it highlights the well-known complexity of the trait and continued efforts are needed for, (i) understanding the genetic basis of GY and stability in different environments and lines, (ii) exploring the value of GS for predicting and recycling parents earlier in the cycle to reduce the cycle time, (iii) predicting the GY potential of crosses and (iv) determining the value of sparse testing, where population sizes can be increased and lines can be tested in fewer sites and environments.

## Data Availability Statement

The genotyping data for 23, 526 lines used in this study has been uploaded to Figshare and is available in: https://doi.org/10.6084/m9.figshare.12350000.v1.

## Author Contributions

PJ planned the study, performed the analyses, and drafted the manuscript. RS, H-JB, JP, and JH-E supervised the work and designed the experiments. LC-H, VG, and SM generated the phenotyping data. SS called the marker polymorphisms.

## Funding

This research was supported by the Delivering Genetic Gain in Wheat (DGGW) project (funded by the Bill and Melinda Gates Foundation and the United Kingdom Department for International Development (DFID) and managed by Cornell University) under the terms of Contract No. OPP1133199 and Feed the Future project through the U.S. Agency for International Development (USAID), under the terms of Contract No. AID-OAA-A-13-00051. The opinions expressed herein are those of the authors and do not necessarily reflect the views of the USAID.

## Conflict of Interest

The authors declare that the research was conducted in the absence of any commercial or financial relationships that could be construed as a potential conflict of interest.
